# Managing Semi-Arid Rangelands for Carbon Storage: Grazing and Woody Encroachment Effects on Soil Carbon and Nitrogen

**DOI:** 10.1371/journal.pone.0109063

**Published:** 2015-10-13

**Authors:** Hasen M. Yusuf, Anna C. Treydte, Jauchim Sauerborn

**Affiliations:** 1 College of Agriculture and Natural Resources, Wollega University, 395 Nekemte, Ethiopia; 2 Institute of Plant Production and Agroecology in the Tropics and Subtropics, University of Hohenheim, 70593 Stuttgart, Germany; Institute for Sustainable Plant Protection, C.N.R., ITALY

## Abstract

High grazing intensity and wide-spread woody encroachment may strongly alter soil carbon (C) and nitrogen (N) pools. However, the direction and quantity of these changes have rarely been quantified in East African savanna ecosystem. As shifts in soil C and N pools might further potentially influence climate change mitigation, we quantified and compared soil organic carbon (SOC) and total soil nitrogen (TSN) content in enclosures and communal grazing lands across varying woody cover i.e. woody encroachment levels. Estimated mean SOC and TSN stocks at 0–40 cm depth varied across grazing regimes and among woody encroachment levels. The open grazing land at the heavily encroached site on sandy loam soil contained the least SOC (30 ± 2.1 Mg ha^-1^) and TSN (5 ± 0.57 Mg ha^-1^) while the enclosure at the least encroached site on sandy clay soil had the greatest mean SOC (81.0 ± 10.6 Mg ha^-1^) and TSN (9.2 ± 1.48 Mg ha^-1^). Soil OC and TSN did not differ with grazing exclusion at heavily encroached sites, but were twice as high inside enclosure compared to open grazing soils at low encroached sites. Mean SOC and TSN in soils of 0–20 cm depth were up to 120% higher than that of the 21–40 cm soil layer. Soil OC was positively related to TSN, cation exchange capacity (CEC), but negatively related to sand content. Our results show that soil OC and TSN stocks are affected by grazing, but the magnitude is largely influenced by woody encroachment and soil texture. We suggest that improving the herbaceous layer cover through a reduction in grazing and woody encroachment restriction are the key strategies for reducing SOC and TSN losses and, hence, for climate change mitigation in semi-arid rangelands.

## Introduction

Soil is the largest terrestrial reservoir of carbon (C) and nitrogen (N) [1] and can store about three times as much C and N than the atmosphere [2], sequestered mainly in decomposed plant litter and residues. Recent rapid losses of soil C and N due to intensive livestock or agricultural uses and changes in fire regimes have been reported for tropical savannas, which cover ca. 10 to 15% of all terrestrial ecosystems [3–4]. These ecosystems, if well managed, may have a high potential to store an appreciable fraction of atmospheric CO_2_ as organic carbon (OC) in the soil [4]. Given the vast area cover of savanna systems, enhanced C and N fluxes from these systems linked to land use and cover changes could greatly influence the global C and N cycle, with direct consequences for potential climate change mitigation and adaptation strategies [2]. Nevertheless,soil C and N dynamics in savanna ecosystems are complex and poorly understood as the impacts of land use and associated vegetation cover changes, climate and soils are complex and vary spatially and temporally.

Previous studies have shown mixed results of grazing effects on soil organic carbon (SOC) and soil organic nitrogen (SON), with studies showing positive [5], neutral [6] or negative effects of grazing [7]. Grazers affect SOC and SON by mechanisms that alter C and N inputs and outputs from the soil [8]. Higher grazing intensities are generally thought to decrease soil C and N by direct removal of aboveground herbaceous biomass, i.e., reduction of potential CO_2_ fixation in photosynthetic tissue and reduction in belowground C inputs through lower root production and higher root litter turnover [9, 10]. Further, grazers can affect legume abundance and hence N fixation rates, which may alter N inputs to the soil [11]. Ruminant enteric fermentation; C and N emissions from animal wastes through volatilization and leaching impact SOC and TSN stock in the soil [12]. Changes in soil C and N outputs associated with grazers arise mainly from changes in soil organic matter decomposition and mineralization rates [13] or increased erosion under grazing [14]. Grazing generally decreases litter and herbaceous plant cover and thus may increase soil organic matter mineralization rates because of greater soil temperature fluctuations and/or soil moisture variability and by increasing desertification [13]. The effect of grazing on SOC and SON stocks depends on precipitation, soil types/texture, plant species composition, and grazing intensity [5–14]. Hence, the overall consequences of grazing on SOC and SON accumulation may vary along gradients of these variables and so far only a few studies have been conducted on quantifying these effects in semi–arid rangelands of east African pastoral grazing systems.

Over the last century, African savannas have been encroached by woody species despite differing climate and management practices such as variable domestic herbivore stocking rates and fire regimes [15]. Woody encroachment, which refers to an increase in cover, density and biomass of indigenous woody plant species, has been reported over much of the world’s arid and semi-arid environments (‘drylands’) in recent decades [16]. The causes of woody encroachment include overstocking, changes in fire frequency and intensity [17], changes in N deposition [18], increasing atmospheric CO_2_ concentration and climate change [19]. Similarly, the structure and composition of semi-arid southern Ethiopian rangeland vegetation has changed dramatically, mainly due to the Ethiopian government`s fire prohibiting policy and grazing intensification since the 1970s [20]. Grazing intensification is an increased degree of grazing by herbivores which is mainly associated with increased stocking density and changes in a traditional pastoral land use system.

Large areas of southern Ethiopian rangelands have become encroached by woody plant species, resulting in a substantial reduction of the herbaceous layer and, as a result, of pastoral productivity [21]. While woody encroachment is often regarded as severe rangeland degradation, particularly in the context of cattle grazing or pastoral production [21], it was also shown to trigger a large increase in C sequestration potential in tropical America [22], Australia [23], and South African [24] savanna systems. This C accumulation appears to be a function of enhanced below- and aboveground net primary productivity (NPP), low decomposition rates beneath shrubs, biochemical recalcitrance of shrub litter, and organic matter stabilization in protected soil aggregates [23]. However, this seems to be precipitation-dependent, i.e., the drier sites in the Chihuahuan desert in USA (< 280 mm rainfall) gained soil C and N with encroachment while more mesic sites (>600 mm rainfall) lost C and N with encroachment [25].So far, little quantitative information is available on soil C and N stocks capacities and the influence of woody encroachment on these stocks in east African semi-arid savanna systems.

A recent assessment of aboveground vegetation biomass and cover data has indicated an increasing potential for aboveground C stocks by encroaching woody plant species in a semi-arid Ethiopian rangeland [26]. However, it is not yet clear how the influence of this woody encroachment has affected the soil C sink and the soil C influx that arises when grazing pressure is relaxed. This information is critically important since more than 70% of the ecosystem C pool is located in the soil [2] and could potentially be influenced by intensification of grazing and vegetation cover changes.

The main aim of this study was to investigate whether soil C and N stocks have increased with woody encroachment and how this dynamics interacts with grazing practices. We expect that severely woody encroached sites will contain the most SOC and total soil nitrogen (TSN) stocks. We further would expect that long-term grazing relaxation (rangeland enclosure) will increase SOC and TSN stocks. Thirdly, we hypothesize that woody encroachment and grazing exclusion will interact and that, as a result, severely encroached sites from which grazing has been excluded for a long time would have greater SOC and TSN than sites that are less encroached and grazed.

## Material and Methods

### Ethics statement

The permit for soil and plant sampling was obtained from the Yabello District Agricultural Office and Village level pastoral community leaders, which are responsible for the management of the communal rangelands of Borana pastoral community.

### Study area

Study sites were located in a semi-arid pastoral system within approximately 10–70 km apart in the Yabello and Dire Districts, Borana, southern Ethiopia. This semi-arid rangeland is used predominantly for livestock (cattle, camel, goat and sheep) production [27]. The sites represent similar soil types, climatic conditions and livestock population densities, but vary considerably in vegetation cover, elevation and topography (Tables 1 and 2). The soil in the study sites is Chromic Cambisol according to the FAO/UNESCO system (unpublished data). The study sites are encroached by woody vegetation, with < 40% of the shrubs established before 1970s [28], and the most extensive woody encroachment occurring after the 1980s because of increased grazing pressure and fire suppression [20].

**Table 1:** Woody encroachment levels, age of enclosure, soil and livestock population density characteristics of the research sites in the Yabello and Dire districts of Borana zone. Geological information was summarized from the Borana land use study project soil survey report (unpublished data).Woody encroachment levels:Low woody encroachment site (LE), moderate woody encroachment site (ME), severe woody encroachment site (SE), highest woody encroachment (HE).

Site (local names)	Encroachment level	Location (latitude, longitude)	Elevation (masl)	Geology	Soil type	Enclosure age	Soil texture (%)			Textural class	Livestock (head km^-2^)					*LU km ^-2^
							Sand	Silt	Clay		Cattle	Camel	Goat	Sheep	equines	
Did -yabello	LE	04^0^ 56^´^ 33^´´^	1542–1564	Quartz-feldspathic gneiss and alluvium (sand silt and clay)	Cambisols	35	46	10	44	Sandy Clay	30	1.05	11	3	0.7	23.9
		38^0^ 10^´^12^´´^														
Damballa—badana	ME	04^0^ 24´02^´´^	1439–1514	Alluvium: sand, silt and clay	Cambisols	30	67	18	15	Sandy Loam	31	0.7	9	10	2	25.6
		38^0^ 17^´^ 03^´´^														
Massade	SE	04^0^ 41^´^ 08^´´^	1436–1518	Quartz-feldspathic gneiss	Cambisols	8	65	20	15	Sandy Loam	25	2.6	18	12	0.5	23.3
		38^0^ 1141^´´^														
Argaassa	HE	04^0^ 38^´^ 58^´´^	1247–1323	Plateau basalt: alkaline basalt and trachyte	Cambisols	12	64	19	17	Sandy Loam	25	2.6	18	12	0.5	23.3
		38^0^ 04^´^ 58^´´^														

* Livestock Unit (LU) was calculated using the livestock population data obtained for each site from local Agricultural Offices following [30].

1 LU = 250 kg live weight.

**Table 2:** Mean values (± SE) of vegetation characteristics of the grazing regimes for each encroachment level in the Yabello and Dire districts of Borana, southern Ethiopia. Low woody encroachment site (LE), moderate woody encroachment site (ME), severe woody encroachment site (SE), highest woody encroachment site (HE). Open = open access grazing land, enclosure = areas of livestock exclosure, reserved for heifers and calves only in the dry season. N = number of plots sampled in each grazing regime.

Variables		LE			ME			SE			HE		
	N	Open	Enclosure	Δ	Open	Enclosure	Δ	Open	Enclosure	Δ	Open	Enclosure	Δ
Tree canopy cover* (%)	20	0**±0**	10**±2**	10	19**±4**	7**±2**	-12	46**±8**	18**±7**	-28	16**±7**	20**±7**	4
Shrub canopy cover (%)	20	9**±2**	34**±7**	25	40**±11**	45**±9**	5	23**±5**	36**±9**	13	48**±11**	60**±9**	12
**Total woody canopy cover (%)**	**20**	**9±2**	**44±5**	**35**	**59±13**	**52±10**	**-7**	**69±9**	**54±11**	**-15**	**64±14**	**80±11**	**16**
**Herbs canopy cover**	**20**	**70±12**	**62±9**	**-8**	**72±15**	**71±11**	**-1**	**24±6**	**62±11**	**38**	**51±16**	**67±15**	**16**
Tree density ha^-1^	20	0**±0**	74**±13**	74	111**±17**	32**±9**	-79	200**±55**	116**±61**	-84	170**±47**	214**±43**	44
Shrubs density ha^-1^	20	616**±117**	1532**±179**	916	1732**±189**	2368**±135**	636	865**±**61	1215**±73**	350	1425**±137**	1895**±95**	470
**Total density**	**20**	**616±117**	**1606±224**	**990**	**1843±217**	**2400±139**	**557**	**1065±78**	**1331±92**	**266**	**2065±195**	**2109±123**	**44**

* Canopy cover refers to the proportion of the ground area covered by the vertical projection of the tree/shrub/herb canopy.

Δ = % canopy cover or density difference between enclosures and open grazing.

Fifty years (1957–2012) of climate data (Ethiopian Metrological Agency) indicated a long-term mean annual precipitation of 550 mm in the region, with a 66% coefficient of variation across years. Rainfall has a bimodal distribution, with 55% of the annual precipitation occurring in March—May, followed by 30% in September—November. Mean annual air temperature is 20°C, with a mean monthly maximum of 21°C in February and a mean monthly minimum of 18.5°C in July.

### Land use and grazing patterns in Borana

Historically, the land use system in Borana was largely characterized by sustainable exploitation of rangeland resources based on herd mobility associated with flexible stocking densities [28]. Movement patterns corresponded with local rainfall and associated natural resource productivity, shifting towards dry areas in the wet season and more humid areas in dry seasons [28]. The land use also involved periodic burning of the rangelands [28]. Following the 1970s drought period in the area, several ponds or deep wells were established in some parts of the rangelands and the pastoralists shifted to use the areas near these ponds or deep-wells (permanent water points) for grazing in the dry season and drought years, whereas the other parts of the landscape were utilized during wet season [29]. However, this extensive, the season based rotational grazing system has changed to a semi-sedentary year-round intensive grazing system since the 1980s because of increasing human and livestock populations, water points, roads and market infrastructure development, settlement programs and frequent drought events [20]. The Borana pastoral community was estimated to be 480,000 people in 1980s, increasing with an annual population growth rate of about 2.5–3% [31].

The livestock density in 1982, measured by aerial observation, was 14.3 and 11.9Tropical Livestock Unit (TLU) km^-2^(1TLU = 250 kg live weight)[30] domestic herbivore stocking rates in the wet and the dry-season, respectively[32]. By 2000, a household based survey provided stocking densities of 45–153 TLU km^-2^[33]. Similarly, Homann et al., [2008] estimated 105 and 43 TLU km^−2^ during and after the 1999/2000 drought year, respectively [31]. Though the livestock population increase is often dampened by frequent drought events (occurring every 5to 6 years), a rise in the net livestock density beyond stocking carrying capacity has been reported in the Borana rangelands[31,33].

More exclusive forms of land ownership have been introduced since the mid-1960s by the establishment of traditional rangeland enclosures and government ranches [33]. The former is a small section of grazing land put aside during the wet season by individual pastoral households or the community to conserve pasture for calves, heifers, and sick animals during the dry season. Fires have been completely suppressed by pastoralists in the rangelands since the 1970s because of government regulations and because the standing biomass was rather used for forage, to support high cattle densities [28].

By 1980s, with the expansion of ponds, boreholes and shallow wells and government settlement programs, crop cultivation has drastically expanded into wetter and more valuable grazing areas [31]. By 2000, more than 16% of the total grazing area had been converted to crop cultivation [33]. Year-round intensive grazing combined with suppression of fire and other climatic factors led to the conversion of grass into shrub-dominated savanna/woodlands [20].

### Sampling design

**Fig 1:**
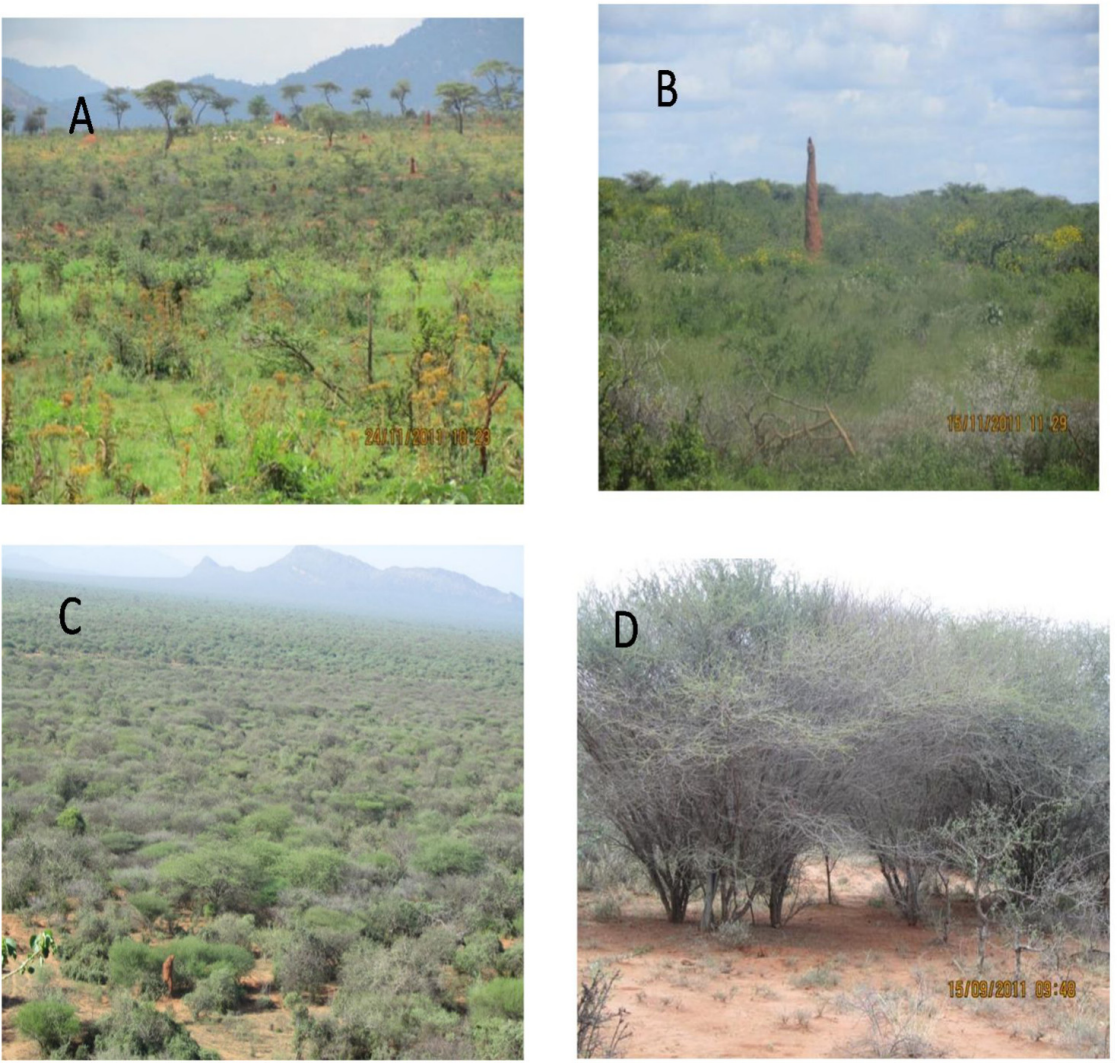
Study sites: low woody encroachment site (A), moderate woody encroachment site (B), severe woody encroachment site (C), highest woody encroachment site (D) in the semi-arid rangelands of Yabello and Dire districts, Borana, southern Ethiopia. Pictures taken by Hasen-Yusuf in 2011.

The study was conducted along a gradient of woody plant encroachment, representing four levels of woody encroachment in southern Ethiopian pastoral rangelands. The levels were based on the stage of woody encroachment determined through personal interviews with local people and district agricultural office managers, and also supported by ground quantification of the woody plant canopy cover and density (Table 2). Woody vegetation structure was quantified by measuring tree/shrub density; canopy diameters, canopy height, and stem height of the woody species using an 8-m long graduated wooden pole. Canopy cover was calculated using the average of the two longest canopy diameters perpendicular to each other and parallel to the ground. Stem height was measured as the total height of the plant stem from the ground level to the highest foliage. These data were used to compute tree and shrub densities and canopy cover per hectare for the grazing regimes and encroachment levels (Table 2). The woody encroachment levels were arranged from lowest to highest encroachment. The site of low encroachment (LE) has a mosaic of tree and shrub patches in a perennial herbaceous species stand, with an average total woody canopy cover of 27%. The site with moderate encroachment (ME) has dwarf shrubs and thick perennial grass dominated stands with an average total woody canopy cover of 56%. The site with severe encroachment (SE) contains fully matured tree and shrub stands with a woody canopy cover of 62%, in which herbaceous plants have been almost eliminated. The site with the highest level of encroachment (HE) has small to medium-sized shrubs and trees that form an almost impenetrable thicket with a canopy cover of 72% (Table 2, Fig 1).

**Fig 2:**
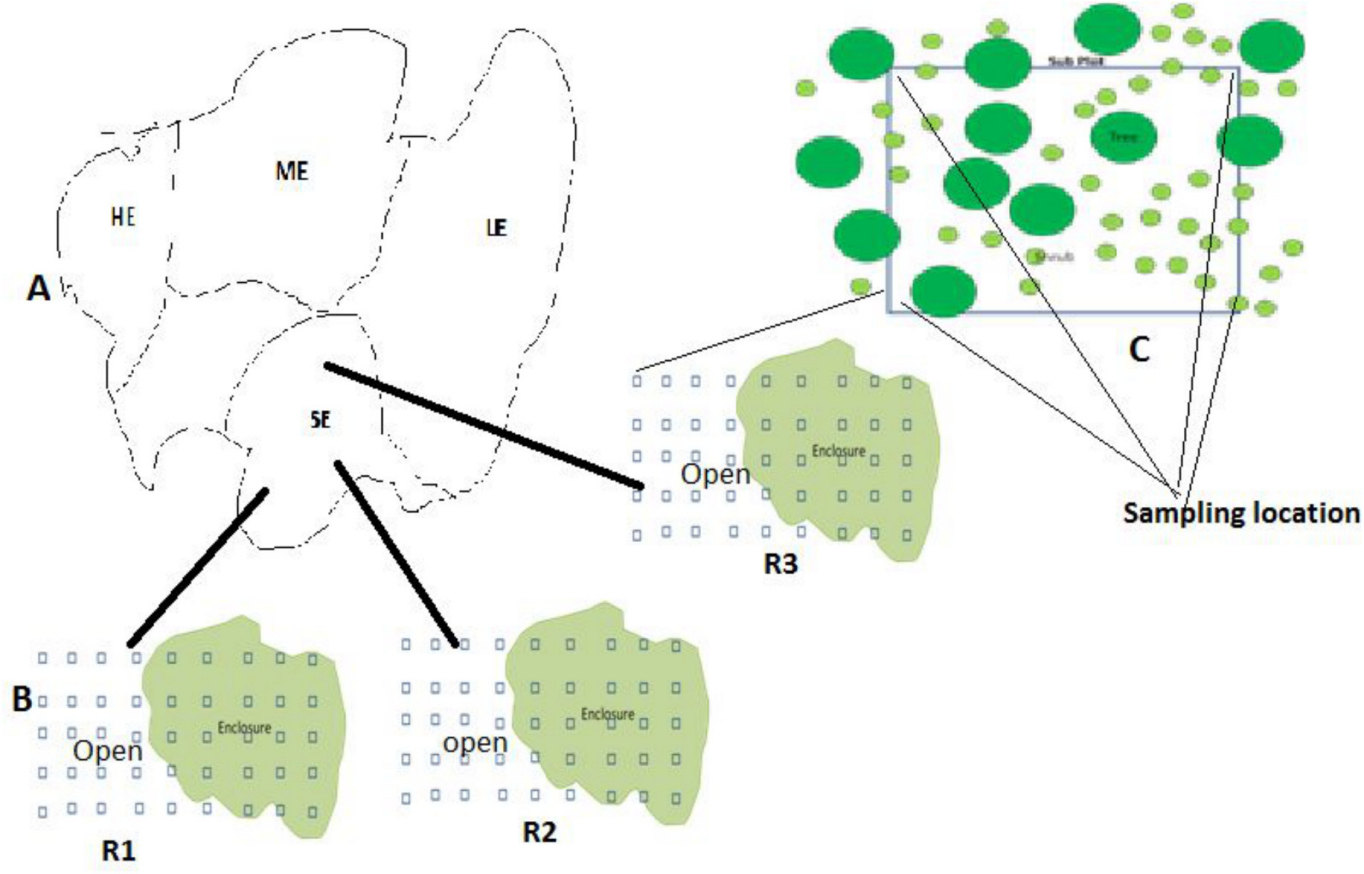
Schematic map of the location of the four levels of woody encroachment (panel A: Low woody encroachment site (LE), moderate woody encroachment site (ME), severe woody encroachment site (SE), highest woody encroachment site (HE), transects and plots in the open and enclosure grazing regimes (B) and their replications (R1, R2 and R3) and soil sampling location at each sub plots(C).

Each woody encroachment level was divided into two grazing regimes namely, limited grazing (hereafter named ‘enclosure’) and communal grazing land (hereafter named ‘open’) (Fig 2).The open grazing land represents the most common land use system in the Borana rangelands and is defined as the communal rangelands that are not privately owned, yet belonging to the communities whose members have equal access rights to the communal resources [34]. Enclosures in this study represent a fenced area that covers10–25 ha grazing land and protects from grazing during the wet season, although some grazing may occur in the enclosure in the late dry season and in drought years when the forage is extremely scarce [35].

We randomly selected three replicates of both grazing regimes within the same age group and 10–25 ha in size and 2–5 km apart (aerial distance, measured using Garmin GPS 72 (Garmin International Inc., USA, Fig 2). The replicates in each site were located on similar lithology, soils, topography and slope. To measure soil and vegetation attributes within each grazing regime we established three belt-transects (10 m width×1000 m length), 300–500m apart. Along each transect, four (10 × 10 m) plots were established at 200 m intervals along the linear belt transect using meter tape, GPS and compass, bringing the total number of plots sampled to 288 plots (4 woody encroachment level × 2 grazing regime × 3 replicate grazing regimes × 3 belt transects × 4 plots).Previous studies in the present study area have shown that plot sizes < 100 m^2^ were effectively used for sampling shrub dominated vegetation [36]. To assess herbaceous species composition, biomass, and diversity inside and outside the enclosures, caged subplots of (1m x 1m = 1m^2^) were randomly nested within the larger 100 m^2^ plots used for woody species.

### Sampling and laboratory analysis

Vegetation sampling was done at the end of the long and short rainy seasons (end of May and mid December, respectively), soil samples were collected at the end of the long rainy season. Herbaceous aboveground biomass was destructively quantified [37]; grass and forb species rooted within the caged 1 m^2^ subplot were harvested to ground level, oven dried (at60°C for 48 h) and weighed using a 0.1 g scale.

Four soil core samples at 0–20 and 21–40 cm depth were collected from each corner of the subplots using an intact soil core sampler of 6.5 cm diameter and using the intact soil core sampling method[38]. Samples of the same depth were mixed thoroughly in a large bucket in order to obtain one composite soil sample per depth increment [38]. The soil samples of the depth increments excavated in pits were removed from the hole and extracted before the core was augered to the next depth increment to minimize compaction of each depth increment. The device also allowed estimating the bulk density of each soil depth increment from intact soil core samples [38, 39]. Soil cores were placed in plastic lined paper bags and oven dried (107°C) until constant weight [40]. Cores were sieved through a 2 mm sieve, and fine soil (< 2 mm), coarse roots (> 2 mm), and gravel (> 2 mm) were separated and weighed to the nearest 0.1 g. Coarse herbaceous roots and other belowground organic material were separated both visually and by flotation methods from soils, rocks, and gravel and oven dried (60°C) to constant weight [39]. The carbon (C) content of the above- and belowground vegetation biomass was estimated as 47% of the dry mass [41]. The fine soil (< 2 mm) fraction was then ground using an analytical mill (IKA^®^, Model A10) [39]. A fraction of the soil samples were treated with 0.1 M HCl before analysis to test for inorganic carbon. Samples which tested positive for inorganic C were completely digested with 0.1M HCl to remove inorganic C [39]. Standard analytical procedures of the Ethiopian National Soil Testing Center were used for all chemical and physical analyses. SOC was determined using the Walkley–Black method [42] and TSN was determined using Kjeldahl [43]. Ammonium and sodium acetate extracts were used to determine exchangeable cations (EC) and cation exchange capacity (CEC) [44], pH and electric conductivity (EC) were determined using a suspension of 1:5 soil:water. Particle size analyses were done using the Hydrometer method [45]. Bulk density (g m^-3^) was calculated as the mass of the fine soil (<2 mm) fraction divided by the volume of the entire core to avoid overestimating the mass of the soil when stones and gravels were present [46]. Percent SOC and TSN were multiplied by each sample fraction mass to obtain total SOC and TSN per core sample [39]:

$$SOC\;\left(kg\;ha^{\text{-}1}\right)=Mass<\;2mm\;soil\;\left(kg\right)\;/\;Volume\;of\;core\;\left(cm^{3}\right)\times d\times cf\times C$$ (1)

$$TSN\;\left(kg\;ha^{\text{-}1}\right)=Mass<2mm\;soil\;\left(kg\right)/Volume\;of\;core\;\left(cm^{3}\right)\times d\times cf\times N$$ (2)

Where *d* = depth (cm), *C* = organic carbon concentration, *N* = total nitrogen concentration and *cf* is the conversion factor = (kg cm^-3^) × (10,000 cm^2^ m^-2^) × (10,000 m^2^ ha^-1^).

### Statistical analyses

The influences of grazing, woody encroachment and soil depth and their interactions on SOC % and SOC stock, herbaceous root OC content, TSN % and total TSN stock, SOC:TSN ratio, and soil bulk density were evaluated using SAS version 9.1 mixed model procedures (Proc MIXED). Differences in all response variables were evaluated by treating woody encroachment level as main effect, grazing management regime and soil depth was nested within woody encroachment level, age of enclosures was considered as random effect. Mean comparisons were made using Tukey’s test (p < 0.05). All values reported are means (± SE). Linear regressions were used to determine the relationship between SOC and TSN concentration, soil texture, CEC, pH, and soil bulk density.

## Results

### Carbon stock in the herbaceous vegetation

Significant differences between herbaceous aboveground C (Hag OC) and root stocks (HR OC) were found between open and enclosure plots with the exception of the LE site which had lower stocks in a comparison to the other sites (Table 3). However, mean herbaceous aboveground -and root C stocks did not show a consistent trend of decrease or increases with the increase in the levels of woody encroachment. More than 85% of herbaceous root biomass C storage was found in the top 20 cm soil depth and its vertical distribution in the 21–40 cm soil depths was not significantly affected by grazing management and woody encroachment (Table 3).

**Table 3:** Mean (± SE) herbaceous root biomass carbon (HROC) and herbaceous aboveground biomass C (Hag OC) in enclosures and open grazing land across four levels of woody encroachment. Encroachment levels: Low woody encroachment site (LE), moderate woody encroachment site (ME), severe woody encroachment site (SE), highest woody encroachment site (HE). N, grazing regime replicates per site. Different lowercase letters represent statistical differences determined by the interaction of grazing regime and woody encroachment terms in the mixed Model andTukey’s means comparisons (P < 0.05).

Encroachment level	Grazing regime	N	HR OC (Mg ha^-1^)			Hag OC (Mg ha^-1^)
			0–20cm	21–40cm	Total	
LE	Open	3	0.49±0.10	0.03±0.02	0.52±0.11^a^	0.37±0.03^a^
	Enclosure	3	0.20±0.20	0±00	0.29±0.47^a^	0.29±0.03^a^
ME	Open	3	1.01±0.32	0.04±0.04	1.06±0.35^b^	0.97±0.06^b^
	Enclosure	3	1.53±0.43	0.08±0.05	1.61±0.38^c^	1.65±0.15^c^
SE	Open	3	0.47±0.26	0.08±0.08	0.54±0.27^a^	0.03±0.01^d^
	Enclosure	3	2.11±0.88	0.26±0.13	2.37±0.68^d^	0.17±0.01^a^
HE	Open	3	0.50±0.45	0±0	0.50±0.44^a^	0.55±0.06^a^
	Enclosure	3	1.84±0.51	0.12±0.07	1.96±0.38^cd^	0.90±0.06^a^
Encroachment					NS	**
Grazing					NS	*
Depth					**	-
Encroachment x depth					NS	-
Encroachment x grazing					*	*
Grazing x depth					NS	-

** highly significant at p <0.01

*significant at p < 0.05

NS not significant at p ≥0.05.

### Soil organic carbon and nitrogen

**Fig 3:**
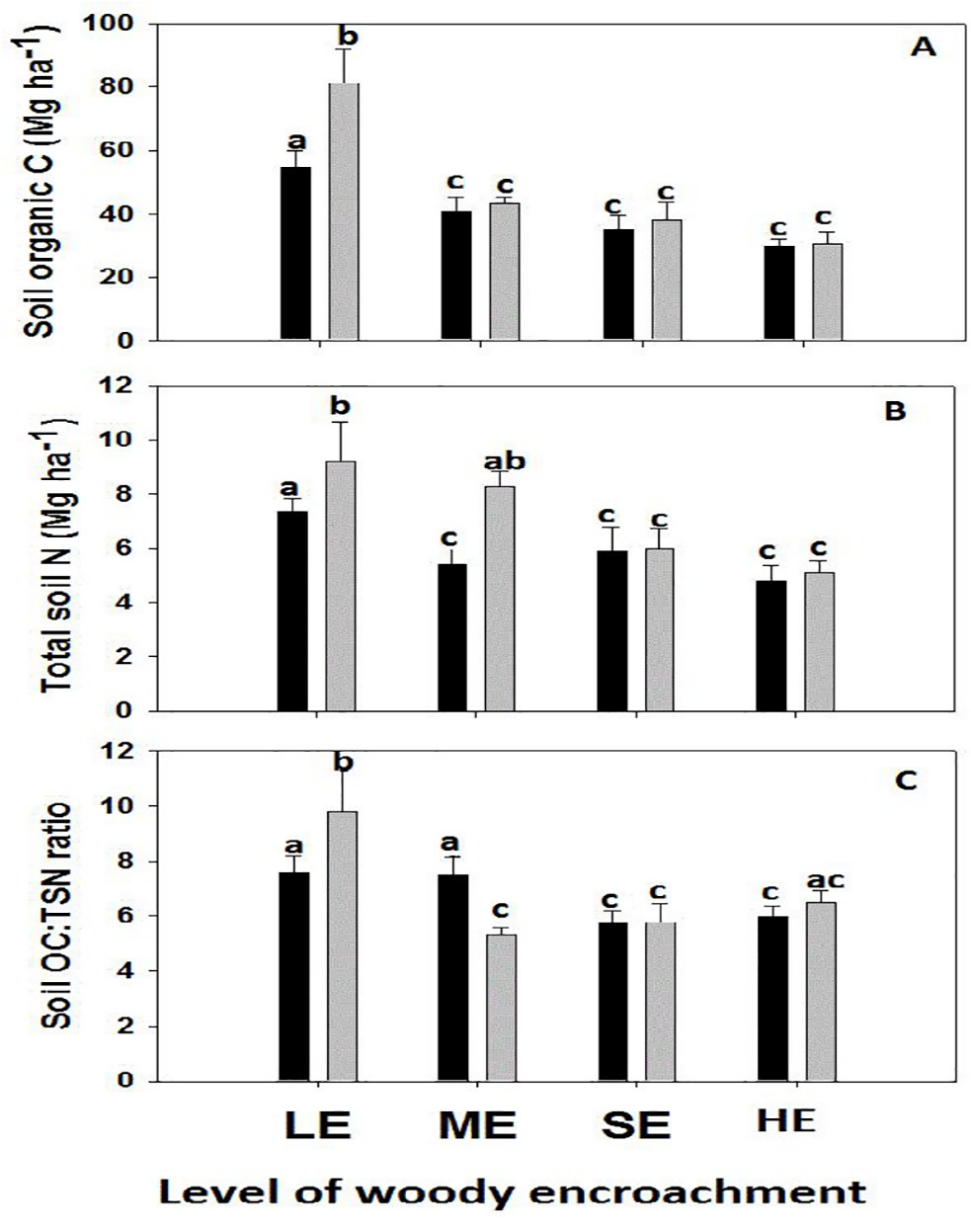
Means and standard errors by levels of woody encroachment and grazing regime (grey bars = enclosure, black bars = open grazing land) for soil organic carbon (A), total soil nitrogen content (B), and soil organic carbon to total soil nitrogen ratio (C).Low woody encroachment site (LE), moderate woody encroachment site (ME), severe woody encroachment site (SE), highest woody encroachment site (HE). Different lowercase letters represent statistical differences determined by woody encroachment by grazing interaction term in themixed model and Tukey’s means comparisons (P < 0.05).

The mean total SOC stock and TSN stock in 0–40 cm soil depth ranged from 29.8 ± 2.35 and 4.8 ± 0.56 Mg ha^-1^respectively, in the open grazing soils at the HE site to 81.0 ± 8.04 and 9.2 ± 1.1.32 Mg ha^-1^ respectively, in the enclosure soils at the LE site (Table 4), with low variances except at LE (Fig 3A and 3B).

**Table 4:** Mean (x SE) soil bulk density (BD), percent soil organic carbon (SOC %), soil organic carbon stocks (SOC), percent total soil nitrogen (TSN %), total soil nitrogen stock (TSN) for two soil depths in enclosures and open grazing land across four levels of woody encroachment (for characteristics see also Table 1). Encroachment levels: Low woody encroachment site (LE), moderate woody encroachment site (ME), severe woody encroachment site (SE), highest woody encroachment site (HE). N, grazing regime replicates per site. Different lowercase letters represent statistical differences determined by the grazing regime and woody encroachment interaction term in the mixed Model andTukey’s means comparisons (P < 0.05).

Encroachment level	Grazing regime	Depth (cm)	N	BD (g cm^-3^)	TSN (%)	SOC (%)	SOC:TN	TSN (Mg ha^-1^)	SOC (Mg ha^-1^)
LE	Open	0–20	3	1.3±0.10	0.13±0.01	1.00±0.10	7.6±0.60	3.9±0.27	29.5±2.98
		21–40	3	1.5±0.08	0.13±0.01	0.90±0.09	6.2±0.60	3.5±0.21	24.9±2.53
		**Total**						**7.4**±**0.34** ^**a**^	**54.4**±**5.21** ^**a**^
	Enclosure	0–20	3	1.0±0.04	0.21±0.03	1.85±0.24	9.8±1.50	6.1±1.05	55.6±8.18
		21–40	3	1.1±0.05	0.11±0.02	0.94±0.10	9.1±1.40	3.1±0.43	25.4±2.42
		**Total**						**9.2±1.32** ^**b**^	**81.0±8.04** ^**b**^
ME	Open	0–20	3	1.3±0.07	0.13±0.01	0.97±0.09	7.5±0.63	3.2±0.36	23.1±2,20
		21–40	3	1.3±0.05	0.09±0.01	0.72±0.11	7.9±0.72	2.2±0.23	17.3±2.74
		**Total**						**5.4±0.58** ^**c**^	**40.4±1.54** ^**c**^
	Enclosure	0–20	3	1.3±0.05	0.23±0.01	1.2±0.07	5.3±0.30	5.0±0.33	26.1±1.45
		21–40	3	1.4±0.04	0.15±0.01	0.77±0.02	5.4±0.37	3.3±0.23	17.2±0.69
		**Total**						**8.3±0.36** ^**ab**^	**43.3±2.60** ^**c**^
SE	Open	0–20	3	1.7±0.04	0.12±0.02	0.70±0.11	6.0±0.36	3.5±0.55	20.9±3.15
		21–40	3	1.5±0.04	0.08±0.01	0.47±0.05	5.8±0.24	2.3±0.34	14.1±1.27
		**Total**						**5.9±0.76** ^**c**^	**35.0±3.02** ^**c**^
	Enclosure	0–20	3	1.6±0.03	0.12±0.02	0.70±0.09	6.5±0.64	3.6±0.50	21.4±2.49
		21–40	3	1.8±0.05	0.07±0.01	0.46±0.06	7.6±0.72	2.4±0.23	17.0±2,77
		**Total**						**6.0±0.62** ^**c**^	**38.3±3.27** ^**c**^
HE	Open	0–20	3	1.2±0.03	0.13±0.01	0.73±0.05	6.0±0.40	3.1±0.39	17.3±1,22
		21–40	3	1.4±0.04	0.08±0.01	0.48±0.03	6.3±0.32	1.2±0.17	12.5±0.93
		**Total**						**4.8±0.56** ^**c**^	**29.8±2.35** ^**c**^
	Enclosure	0–20	3	1.3±0.05	0.13±0.02	0.83±0.11	6.48±0.46	3.0±0.29	19.6±2.21
		21–40	3	1.1±0.06	0.07±0.01	0.45±0.04	6.82±056	2.0±0.19	11.1±1,36
		**Total**						**5.1±0.37** ^**c**^	**30.7±1.60** ^**c**^
Encroachment				**	**	**	**	**	**
Grazing				**	NS	**	*	NS	**
Depth				*	**	**	**	**	**
Encroachment x depth				NS	*	NS	*	*	NS
Encroachment x grazing				**	NS	*	*	NS	*
Grazing x depth				NS	NS	NS	NS	NS	NS

** highly significant at p < 0.01

*significant at p < 0.05

NS not significant at p ≥0.05.

Soil OC stock for 0–40 cm was significantly affected by grazing regime, woody encroachment levels and their interactions (Table 4). Total SN stock for 0–40 cm was significantly affected by woody encroachment levels and encroachment level by grazing regime interaction (Table 4). The LE site tended to have higher SOC and TSN stock in the enclosures while the mean SOC and TSN stocks in the enclosure at ME, SE, HE was statistically the same as the adjacent open grazing area (Table 4, Fig 3)

Mean SOC and TSN stocks tended to be higher in the 0–20 cm soil layer, independently of the level of woody encroachment and grazing regime (Table 4). The 0–20 cm soil layer tended to have twice as much SOC and TSN stock in the enclosures at low woody encroachment levels while the mean SOC and TSN stocks in the 0–20 cm at all other treatments were not statistically different from the 21–40 cm soil layer (Table 4).

Soil OC:TSN ratio had been significantly affected by woody encroachment levels, soil depth and interaction of woody encroachment and grazing regime (Table 4) with enclosures showing both higher and lower ratios at the LE and ME sites but remaining relatively unchanged at severely SE and HE sites, despite an increase in percent SOC and percent TSN concentrations (Table 4).

### Soil bulk density

Significant differences in soil bulk density were not found between open and enclosure area with the exception of the LE site which had lower soil bulk density in the enclosures compared to the open area (Table 4). The bulk density tended to be lower in 0–20 than in 21–40 cm soil depths in less woody encroached sites (LE and ME sites), while it was not consistent for severely encroached sites (SE and HE) sites. The bulk density in the 0–20 cm soil ranged from 1.00 ± 0.04 g m^-3^ (in LE enclosure) to about 70% higher values in the enclosure and open grazing land of SE site (Table 4). The deeper soil (21–40 cm) bulk density ranged from 1.10 ± 0.05 g m^-3^ (in LE enclosure) to 1.80 ± 0.05 g m^-3^ (in SE enclosure).

### Factors related to SOC retention

**Fig 4:**
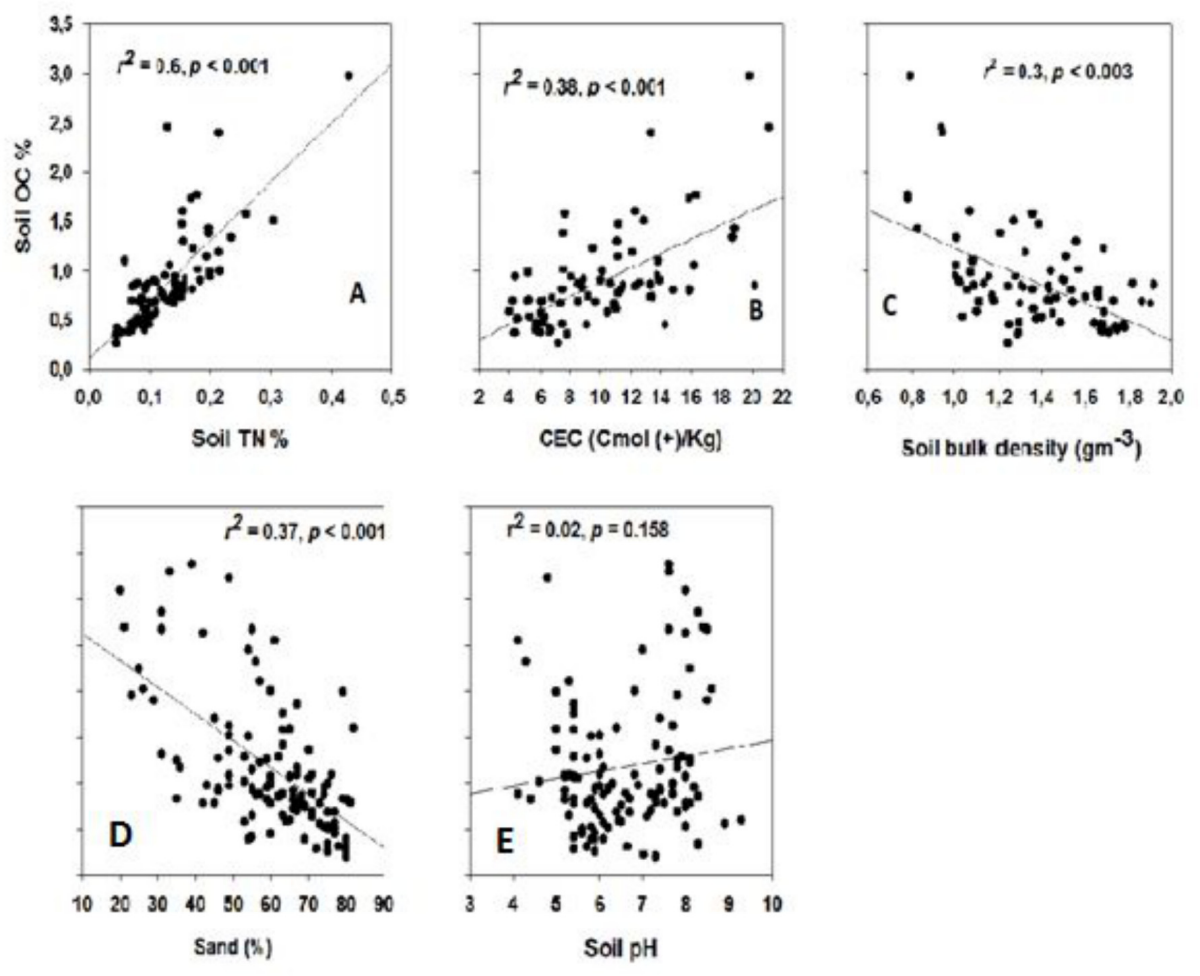
Regression of soil organic carbon fraction (% SOC) against total soil nitrogen fraction (TSN; A), cation exchange capacity (CEC; B), soil bulk density (C), sand fraction (% sand; D) and soil pH (E) within a depth of 0–20 cm. *r* ^*2*^ and *P* values are given for each plate.

Soil OC fractions were linked to the TSN fractions (0–20 cm soil depth) for all study areas (Fig 4A). Soil OC concentration (% SOC) were significantly but weakly negatively related to % soil sand content and bulk density (Fig 4C and 4D).

## Discussion

### Effects of grazing on SOC and TSN stocks

Our results showed that the response of herbaceous above- and belowground biomass C stocks to grazing was strongly influenced by woody encroachment levels. The pattern of the herbaceous above-and belowground biomass C stock response to grazing in most of the woody encroachment sites (e.g., ME, SE, and HE sites) is in agreement with a herbaceous biomass decrease observed in other semi-arid environments [9, 38]. Angassa and Oba, (2010) reported an increase of about 64% in mean herbaceous aboveground biomass in enclosures compared to surrounding open grazing lands after 15–25 years of livestock exclusion in Borana [36]. A more than 200% increase in herbaceous aboveground biomass within the enclosure was also reported from 5–15 year enclosures in northern Ethiopian rangelands [47]. Bagchie and Ritchie (2010) reported a 32–33% increase in aboveground biomass C and a 21–63% increase in root biomass C in livestock enclosures compared to open grazing land in the Trans-Himalayas pastoral system [48]. Schuman et al.(1999) observed a 20–52% and 7–16% increase of C and 15–30% and 18–52% increase of N in aboveground biomass and roots (0–60 cm depth), respectively, after 12 years of livestock exclosure on a native mixed grassland in Wyoming, USA [49]. In our study, however, we found that the effects of grazing on herbaceous above- and belowground biomass C stock were strongly influenced by woody encroachment. In the moderately encroached site, grazing influences on the herbaceous above- and belowground biomass C stocks were strongly visible. However, the lack of significant grazing impacts on herbaceous above- and belowground biomass C stocks at low woody encroachment levels may be associated with the relatively higher woody density and canopy cover when grazing is limited compared to adjacent open grazing land at this site (Table 2) This indicating that woody encroachment could have a significant effect on the expression of grazing in the dynamics of herbaceous above- and belowground biomass C stocks. These results are consistent with other studies that have shown the significant decrease of the understory herbaceous vegetation standing biomass production with an increase in woody density and cover [21, 49]. This decrease may be linked to the competitive advantage of encroacher woody species for soil moisture through their deep root systems and rainfall interception by shrub/tree canopies, which could reduce available soil moisture in arid and semi-arid environments where rainfalls mostly occurs as small events, a response that may increase competitive effects under tree/shrub canopies [50]. Generally, though influenced by woody encroachment, the greater accumulations of herbaceous biomass C in most of our enclosures suggest that there is potential to store more C in the rangelands by reducing grazing pressure.

Our result demonstrated that the response of SOC and TSN to grazing interacted significantly with woody encroachment levels suggesting that the increasing level of woody encroachment and other related site variation (e.g., soil texture) may affect the response of SOC and TSN stocks to grazing. Soil OC and TSN stocks were significantly higher in the enclosures than in the open grazed in low woody encroachment levels on sandy clay soils, but the difference in SOC and TSN was statistically the same in moderate to highest encroachment levels on sandy loam soils. The increase maybe related to increased vegetation (woody and herbaceous) production, litter quality and nutrient cycling [52], and the ability of the soil to retain the extra N after exclusion of herbivory [53]. Our result is in agreement with Mekuria (2013) who reported increased soil organic matter (SOM) and TSN after grazing exclusion for 5–10 years in northern Ethiopia [54]. Similarly, studies from Central Asia, found a significant decrease of SOC and TSN due to intensive grazing in semi-arid environments [7, 55]. Cumulative root biomass not only increases soil C inputs but also N retention within the soil [56, 57] because both organic N and C dynamics are closely linked in the SOM [13]. Hence, the incorporation of N in root tissue and tight cycling within the root zone has been suggested as a mechanism that can reduce N leaching [57].The higher N concentration and TSN stocks in our enclosures might be a result of lower N losses via volatilisation of ammonia and nitrate through animal urine and dung patches and, thereby, an increase in N availability for SOM formation and storage [53]. Higher N losses will decrease N stocks and limit SOM formation and SOC sequestration in the open grazed system [54].

High SOC and TSN (SOM) in the enclosures can also potentially improve soil physical properties such as soil structure and total porosity, which in turn may increase water infiltration rates into the soil [54, 58]. In our study, soil bulk density was lower in the enclosures compared to the open grazing land and the difference was particularly high at low encroachment levels, which may be linked to the fine, sandy clay textured soils of this site. The effect of grazing intensity on bulk density is especially pronounced in wet and fine textured soils[14] as it is susceptible to soil compaction caused by trampling through livestock[14, 58]. Soil compaction potentially reduces water infiltration and increase runoff which often results in decreasing water availability for plant growth [14]. In addition this can lead to loss of top soil and nutrients especially under intense grazing conditions [14, 54]. As a result this can reduce plant productivity and SOC and TSN storage as observed in most of our open grazing lands. Increased soil erosion due to a decrease in vegetation cover associated with continuous, heavy grazing was reported as the main causes for the loss of soil OM in many parts of African and Central Asian grasslands [7, 14, 55].The lack of significant differences in SOC and TSN between the grazing regimes at severely encroached sites (e.g., SE and HE sites) may be the result of both labile and minerals associated OM loss in the top soil due to livestock trampling induced soil erosion, which amplify the negative effects of heavy grazing on herbaceous productivity and C inputs [59]. Our findings suggest that intensified grazing decreases SOC and TSN stocks, and the losses from the top soil layer can not effectively be restored by short period(< 15 years) grazing exclusion at severely woody encroached sites, particularly on coarse, sandy loam textured soils,which are less resistant to rainfall(e.g., at SE and HE sites).

### Effect of woody encroachment on SOC and TSN stocks

On average, our SOC stocks of about 44Mg ha^-1^fall within the range reported by earlier studies for tropical woodland and savanna ecosystems, i.e.,20–80 Mg ha^-1^[60, 61].Our results demonstrated that SOC and TSN have declined with the increase of woody encroachment level. Several qualitative and quantitative indicator data that we collected at HE and SE sites included (i) high bare soil cover, i.e., 40% and 57%, respectively, (ii) low herbaceous (grasses and herbs)ground cover, i.e., 60% and 40%, respectively, (iii) exposed tree roots (pers. obs.), (iv) a similar SOC and TSN-soil depth relationship of upper and lower soil layers, and (v) similar soil bulk density within the top soil (<20 cm soil) of HE and SE sites with the sub soils(> 20 cm soil) at LE and ME sites (Table 3). This may indicate the likely loss of organic matter from the top soils by erosion at the severely shrub encroached sites, suggesting that high woody cover in this semiarid environment could not effectively reduce soil erosion or restore SOC and TSN stocks. Previous study from the same region had shown a 30–61% lower OM in the top 10 cm soil layer in woody encroached sites compared to open grasslands [62]. The finding also concurs with Guo and Gifford (2002) who showed SOC losses when grassland was converted to plantations in New Zealand rangeland systems [63]. Jackson et al. (2002) also reported a decrease in SOC in semiarid grasslands experiencing woody encroachment and associated the reduction in SOC to the incorporation of soil N to aboveground woody plant parts [25]. Hudak et al., (2003) linked the reduction of SOC and TSN at severely woody encroached site in South African dry savanna to a reduction of herbaceous root production caused by woody encroachment [64]. Woody species once established, often outcompete herbaceous species, reducing the herbaceous above- and belowground biomass [51], which may further expose top soil to livestock trampling and rain. Schlesinger et al. (1990) similarly showed that the bare inter-space between woody plants experiences higher temperatures and evapotranspiration, leading to a slow organic N incorporation, denitrification, ammonia volatilization and increased soil erosion [65].

However, several studies have shown that many other biotic and abiotic factors can determine SOC stocks [66]. Soil properties can influence SOC concentrations and the occurrence of woody encroachment itself [67, 68]. For example, Archer et al. (2001) indicated that soil texture strongly influences where *Prosopis* can establish in a southern Texas savanna rangeland [68]. Similarly, Vågen and Winowiecki, (2013) have shown inherent high soil sand fraction strongly limits SOC stocks in East African savanna and woodlands, independently of climatic factors and vegetation type differences [60]. Similarly, our study suggested that soil texture could played an important role for the low SOC and TSN stocks observed in the heavily encroached sites as the sand content independently explained 37% of the variations in SOC and TSN fractions across the sites. High soil sand content is often associated with less adsorption and stabilization of organic matter [66]. Soils with higher clay content may also form tight aggregates that protect SOC from microbes [69].The historical land-use pattern and disturbance, including soil erosion, condition and productivity of the sites before the occurrence of woody encroachment may also influence the variation in SOC and TSN stocks across the sites [70]. Hence, the initial causes of top soil losses due to erosion in severely woody encroached sites may stem from long-term overgrazing and livestock trampling rather than woody encroachment given the common notion that the latter has been considered a symptom of grazing pressure induced rangeland degradation [28]. Further, semi-arid ecosystems generally have extreme rainfall events that can be highly erosive [60]. Therefore, the low SOC and TSN in the severely shrub encroached sites of our study area might be linked to a high prevalence of soil erosion caused by confounding effects of long term grazing, i.e., livestock trampling in addition to the impacts of high shrub cover on understory herbaceous vegetation productivity.

Lack of sufficient replications in our experimental design limits us to isolate the role of woody encroachment for SOC and TSN storage decline at heavily encroached sites in the presence of uncontrolled many potential factors (e.g., soil texture, slope) that could determine the SOC stocks in these semiarid rangelands. On the basis of our data presented here SOC and TSN stocks tended to decrease as a result of the expansion of woody encroachment into semiarid savanna ecosystem. However, as savanna soils, vegetation structure and climate are highly variable and the SOC and TSN storage can be determine by these factors further field studies will be needed to evaluate the large scale net effects of woody plants encroachment and site characteristics on SOC storage in the Borana rangeland ecosystem.

## Conclusion

The results show that SOC and TSN stocks were partly affected by woody encroachment and grazing management but the magnitude of their effect depended on soil sand content. High woody cover did not increase or maintained SOC and TSN stocks at our sites on sandy loam soils. Hence improving the herbaceous layer cover through a reduction in livestock grazing and woody encroachment restriction are the key strategies to maintain SOC and TSN stocks or reduce their losses and, thereby, for climate change mitigation in semi-arid rangelands.
